# A typical case of herpes zoster on lower limb

**DOI:** 10.11604/pamj.2022.42.313.36485

**Published:** 2022-08-25

**Authors:** Amol Madhav Deshpande, Mayuri Amol Deshpande

**Affiliations:** 1 Department of Rachana Sharir, Mahatma Gandhi Ayurved College Hospital and Research Centre, Datta Meghe Institute of Medical Sciences (Deemed to be University) Salod (H), Wardha, Maharashtra, India,; 2 Department of Kayachikitsa, Mahatma Gandhi Ayurved College Hospital and Research Centre, Datta Meghe Institute of Medical Sciences (Deemed to be University) Salod (H), Wardha, Maharashtra, India

**Keywords:** Herpes simplex, herpes zoster, shingles

## Image in medicine

Herpes zoster is caused by varicella zoster virus. After the previous attack of chickenpox, it remains dormant in body and reactivation causes herpes zoster or shingles. There is prodromal of pain for 1 -4 days. Erythema and oedema are followed by formation of groups of vesicles. The crust form in 15 days without scars or minimal scars. Thoracic intercostal nerves and ophthalmic division of trigeminal nerve most frequently affected. Other spinal nerves also involved. A 64-year-old lady come to Outpatient Department (OPD) with vesicles on waist and right thigh with complaint of burning and severe pain in lumbar area. The patient had taken pain killers/anti-inflammatory drugs over the counter but of no use.

**Figure 1:**
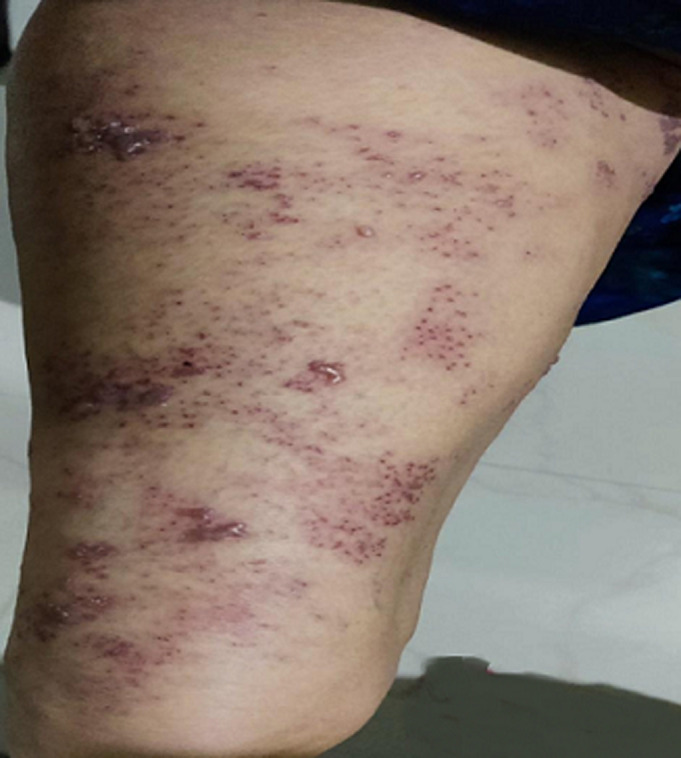
vesicles of herpes zoster on anterior aspect of thigh

